# Psychiatric clinical profiles and pharmacological interactions in COVID-19 inpatients referred to a consultation liaison psychiatry unit

**DOI:** 10.1192/j.eurpsy.2021.290

**Published:** 2021-08-13

**Authors:** N. Arbelo, M. Sagué, H. López-Pelayo, S. Madero, J. Pinzón-Espinosa, G. Anmella, S. Gomes-Da-Costa, L. Ilzarbe, C. Llach, M. Cámara, M.L. Imaz, L. Pintor

**Affiliations:** 1 Department Of Psychiatry And Psychology, Institute Of Neuroscience, Hospital Clinic de Barcelona, Barcelona, Spain; 2 Unit Of Perinatal Mental Health, Hospital Clinic Barcelona, Barcelona, Spain

**Keywords:** COVID-19, Consultation-Liaison Psychiatry, Psychopharmacology, delirium

## Abstract

**Introduction:**

The Coronavirus Disease 2019 (COVID-19) can affect mental health in different ways. There is little research about psychiatric complications in hospitalized patients with COVID-19.

**Objectives:**

The aim of the study was to describe the psychiatric clinical profile and pharmacological interactions in COVID-19 inpatients referred to a Consultation-Liaison Psychiatry (CLP) unit.

**Methods:**

This is a cross-sectional retrospective study, carried out at a tertiary hospital in Spain, in inpatients admitted because of COVID-19 and referred to our CLP Unit from March 17,2020 to April 28,2020. Clinical data were extracted from electronic medical records. The patients were divided in three groups depending on psychiatric diagnosis: delirium, severe mental illness (SMI) and non-severe mental illness (NSMI).

**Results:**

Of 71 patients included (median [ICR] age 64 [54-73] years; 70.4% male), 35.2% had a delirium, 18.3% had a SMI, and 46.5% had a NSMI. Compared to patients with delirium and NSMI, patients with SMI were younger, more likely to be institutionalized and were administered less anti-COVID19 drugs. Mortality was higher among patients with delirium (21.7%) than those with SMI (0%) or NSMI (9.45%). The rate of side effects due to interactions between anti-COVID19 and psychiatric drugs was low, mainly drowsiness (4.3%) and borderline QTc prolongation (1.5%).

**Conclusions:**

Patients affected by SMI were more often undertreated for COVID-19. However, the rate of interactions was very low, and avoidable with a proper evaluation and drug-dose adjustment. Half of the patients with SMI were institutionalized, suggesting that living conditions in residential facilities could make them more vulnerable to infection.

**Disclosure:**

No significant relationships.

